# Can Hypertrophy of the Contralateral Testis Predict the Absence of a Viable Testis in Infancy with Cryptorchidism: A Prospective Analysis

**DOI:** 10.1371/journal.pone.0151528

**Published:** 2016-03-18

**Authors:** Hee Seo Son, Yong Seung Lee, Young Jae Im, Sang Woon Kim, Byung Hoon Chi, Sang Won Han

**Affiliations:** Department of Urology and the Urological Science Institute, Yonsei University College of Medicine, Seoul, Republic of Korea; University of Sydney, AUSTRALIA

## Abstract

This prospective study aimed to evaluate whether Contralateral compensatory testicular hypertrophy (CTH) is valid as a predictive tool for a non-viable testis in children aged between 6 and 18 months, and whether CTH is affected by mini-puberty. Seventy-two testes from 60 boys aged between 6 and 18 months were categorized into three groups: 24 testes contralateral to surgically removed non-viable testes (NVTs), 24 testes contralateral to surgically corrected undescended testes (UDTs), and 24 testes from a normal controls. Contralateral testicular length and volume were measured with ultrasonography and compared among the groups. Group 1 (NVT) had a significantly longer length and larger volume than group 2 (UDT). The length and volume of each group among three developmental periods (6–10, 10–14, and 14–18 months) were also analyzed. In the controls, the length was significantly larger at 6–10 months than at 10–14 months in accordance with previously reported changes in testicular size due to the effect of “mini-puberty.” The volume of controls showed a similar pattern, though without statistical significance. However, this pattern was not observed in the NVT and UDT groups. A receiver operating curve revealed that a testicular length of 16.1 mm or a volume of 0.59 ml had the highest sensitivity and specificity for predicting NVTs. The CTH was also found to be valid as a predictive tool for a NVT in children of ages 6 to 18 months, as the effect of mini-puberty appeared to be absent in the NVT and UDT groups. However, the cut-off values were less than those of previous reports. The proper cut-off level according to the age and measurement method should be applied in this developmental period.

## Introduction

Contralateral compensatory testicular hypertrophy (CTH) in patients with a non-viable testis (NVT) is a well-known phenomenon.[1] The mechanism of CTH is attributable to increased gonadotropin (mainly follicle stimulating hormone [FSH]) secretion associated with positive feedback induced by the undeveloped affected testis.[1, 2] The degree of CTH in terms of the testicular length or volume has been reported to predict the viability of the affected testis in children with a non-palpable testis.[2–6] Hurwitz and Kaptein reported a testicular length of 18 mm in the contralateral testis as a cut-off value for an ipsilateral NVT in children under the age of 11 years,[2] and Shibata et al. reported a cut-off of 2.2 ml of contralateral testicular volume in children under the age of 5 years.[5]

However, for the preservation of fertility, recent guidelines recommend surgical correction of undescended testes as early as possible, starting after 6 months of age and before 18 months of age.[7, 8] Moreover, testicular volume changes due to the unstable level of gonadotropic hormones, also known as mini-puberty,[9, 10] could confuse clinicians using the previous cut-off value during infancy. Therefore, to determine the most appropriate cut-off value for CTH as a predictive tool, an evaluation of these early-aged children is mandatory. We hypothesized that CTH is also valid as a predictive tool for an NVT even in children aged 6–18 months, though with a different cut-off level. To validate this hypothesis, we performed a prospective study in this age group. We additionally analyzed whether CTH is affected by mini-puberty.

## Materials and Methods

### Patients

A prospective study was carried out in accordance with the Declaration of Helsinki after receiving study approval from the Severance Hospital Institutional Review Board (4-2014-0385) and registering the trial at www.clinicaltrials.gov (NCT02203318).

A notice for the clinical study was posted on the bulletin board at our institution, and all participants including controls were voluntarily recruited at their parents’ request. Informed consent was obtained from the parents or legal guardians in written form. Sixty boys aged between 6 and 18 months, who visited our tertiary referral hospital between June 2014 and April 2015 due to cryptorchidism, were enrolled in this study. A total of 72 testes, including 24 contralateral testes (24 patients) from those with a surgically removed unilateral NVT (group 1), 24 contralateral testes (24 patients) from those with a surgically corrected unilateral undescended testis (UDT; group 2), and 24 testes (12 patients) from normal controls (group 3), were included in this study. The categorization of groups 1 and 2 was only made with the operative findings. NVT was diagnosed when a blind-ending spermatic vessel was identified in the abdomen, inguinal canal, or scrotum during operation and was also confirmed histopathologically after excision. Thus, a testis was categorized into group 2 when a viable UDT was found during an operation, even when the diagnosis of a non-palpable testis was made before the operation. Exclusion criteria were: having a known chromosomal abnormality; history of hormone therapy; history of a previous surgery in either the abdomen, inguinal, or genital area; history of a urinary tract infection; retractile testis; or history of epididymo-orchitis.

### Study design

Using a pairwise comparison method, we determined the sample size necessary to achieve statistically reliable results with the minimal number of participants using Shibata et al.’s study,[5] which had reported mean testicular volumes of 2.20 ml and 1.10 ml for the NVT and UDT groups, respectively. Standard deviation was conservatively assumed to be 1.0. According to a two-sided, two-sample equal-variance t-test, a sample size of 24 for each group was obtained. The sample size achieved 90% power to reject the null hypothesis of equality with a standard deviation of 1.0 and a significance level (alpha) of 0.017. An alpha value of 0.0167 (0.05/3 = 0.0167) was conservatively selected to control for type I error from three pairwise comparisons [group 1 (NVT) vs. group 2 (UDT), group 2 (UDT) vs. control, and control vs. group 1(NVT)]. Testicular length and volume were compared among the groups. To verify the presence of size changes during the infantile period, we subdivided each group into three subgroups by age (6–10 months, 10–14 months, and 14–18 months, respectively) and made a comparison of the testicular lengths and volumes.

### Testis size and volume measurement

We measured testicular size with ultrasonography, with the children lying in a supine position with spread legs.[9] The testicular volumes were calculated using a mathematical formula to measure the volume of an ellipsoid: length × width × height × (π/6).[9] To decrease the interobserver variability, measurement in this study was performed by single investigator (HSS).

### Statistical analysis

Categorical and continuous variables were compared using Fisher’s exact test and the Kruskal-Wallis test, respectively. To compare the testicular volumes and lengths among the three groups, a *post hoc* evaluation was conducted using the Mann-Whitney U test with Bonferroni correction. As the 24 control testes were from 12 control patients, we performed a sensitivity analysis that also used the Mann-Whitney U test with Bonferroni correction to determine that each testis was independent from the others. To establish the cut-off value predicting a NVT, a receiver operating characteristic (ROC) curve was constructed. Statistical significance was defined when the p value was less than 0.05. These statistical analyses were conducted with SPSS software, version 18.0 (IBM Corp., Armonk, NY, USA).

## Results

Among the 31 patients with a non-palpable testis, viable UDTs were confirmed during laparoscopy and inguinal exploration in 7 patients. These 7 patients were categorized into group 2, along with 17 patients with palpable UDTs located in the inguinal canal.

### Compensatory testicular hypertrophy in 6- to 18-month-old infants

The median age at evaluation was 11.5 (inter-quartile range [IQR], 8.8–14.4) months. The median age was not different among the three groups (p = 0.358; Table 1).

**Table 1:** Characteristics of 72 testes in 60 patients.

Variable			Group 1/NVT (24 testes in 24 patients)	Group 2/UDT (24 testes in 24 patients)	Group 3/Control (24 testes in 12 patients)	p Value
Laterality (R:L)			17:7	13:11	12:12	0.322
Palpable testis			0	17	24	<0.001
Median age at evaluation (months) (IQR)			12.7 (9.5–14.4)	10.9 (8.2–13.3)	11.7 (8.3–16.6)	0.381
Age distribution in each group						0.835
	6–10 months		7	11	8	
	10–14 months		9	7	8	
	14–18 months		8	6	8	
Median length (mm) (IQR)						
	6–18 months		17.8 (16.1–18.9)	14.7 (13.4–16.0)	13.5 (11.0–15.6)	<0.001
		6–10 months	16.2 (15.7–17.7)	14.4 (13.3–16.0)	15.7 (13.5–15.8)	0.055
		10–14 months	18.0 (16.6–18.6)	14.6 (14.0–16.2)	11.2 (10.7–14.8)	0.001
		14–18 months	19.1 (15.7–20.2)	15.5 (12.5–16.4)	12.8 (10.7–15.0)	0.007
Median volume (ml) (IQR)						
	6–18 months		0.82 (0.66–1.07)	0.53 (0.39–0.61)	0.44 (0.35–0.59)	<0.001
		6–10 months	0.71 (0.61–0.89)	0.53 (0.39–0.58)	0.58 (0.46–0.80)	0.053
		10–14 months	0.79 (0.60–0.98)	0.52 (0.47–0.58)	0.36 (0.30–0.54)	0.008
		14–18 months	0.99 (0.84–1.17)	0.57 (0.34–0.72)	0.42 (0.35–0.58)	0.003

NVT: non-viable testis; UDT: undescended testis; IQR: interquartile range; Categorical and continuous variables were compared using Fisher’s exact test and the Kruskal-Wallis test, respectively.

**Fig 1:**
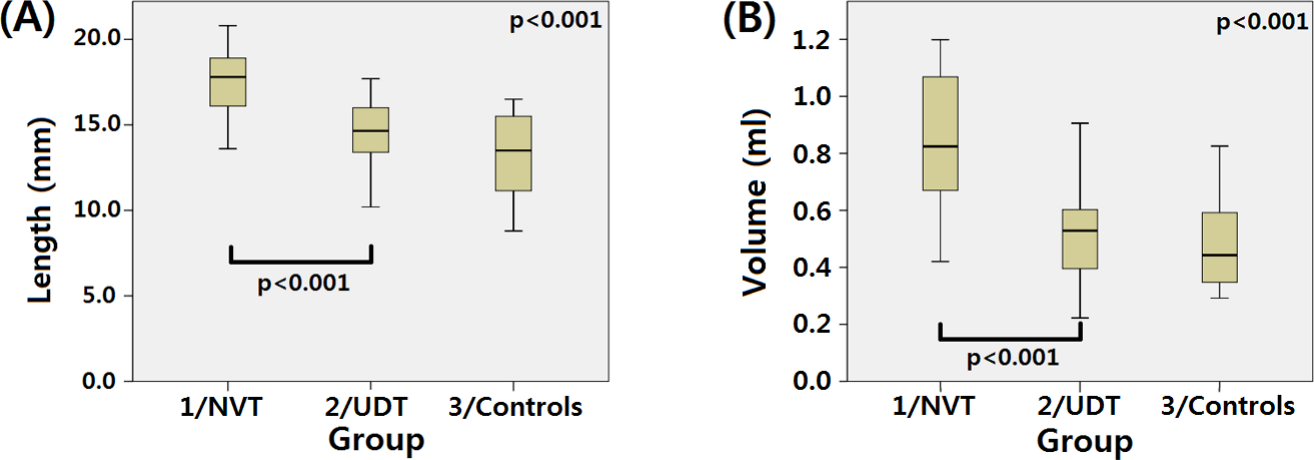
Median testicular length (A) and volume (B) were compared and showed significant differences among the groups. *Post hoc* evaluation using the Mann-Whitney U test revealed that group 1 (NVT) had a significantly longer length and larger volume than group 2 (UDT).

The median testicular length and volume were significantly different among the groups (p < 0.001 and p < 0.001, respectively). A *post hoc* evaluation using the Mann-Whitney U test revealed that group 1 (NVT) had a significantly longer length and larger volume than group 2 (UDT; p < 0.001 and p < 0.001, respectively; Fig 1A and 1B). After a sensitivity analysis of the mean testicular length and volume of each of the 12 controls, we found the same pattern as with the 24 control testes.

### Testicular size changes at 6–18 months of age in each group

**Fig 2:**
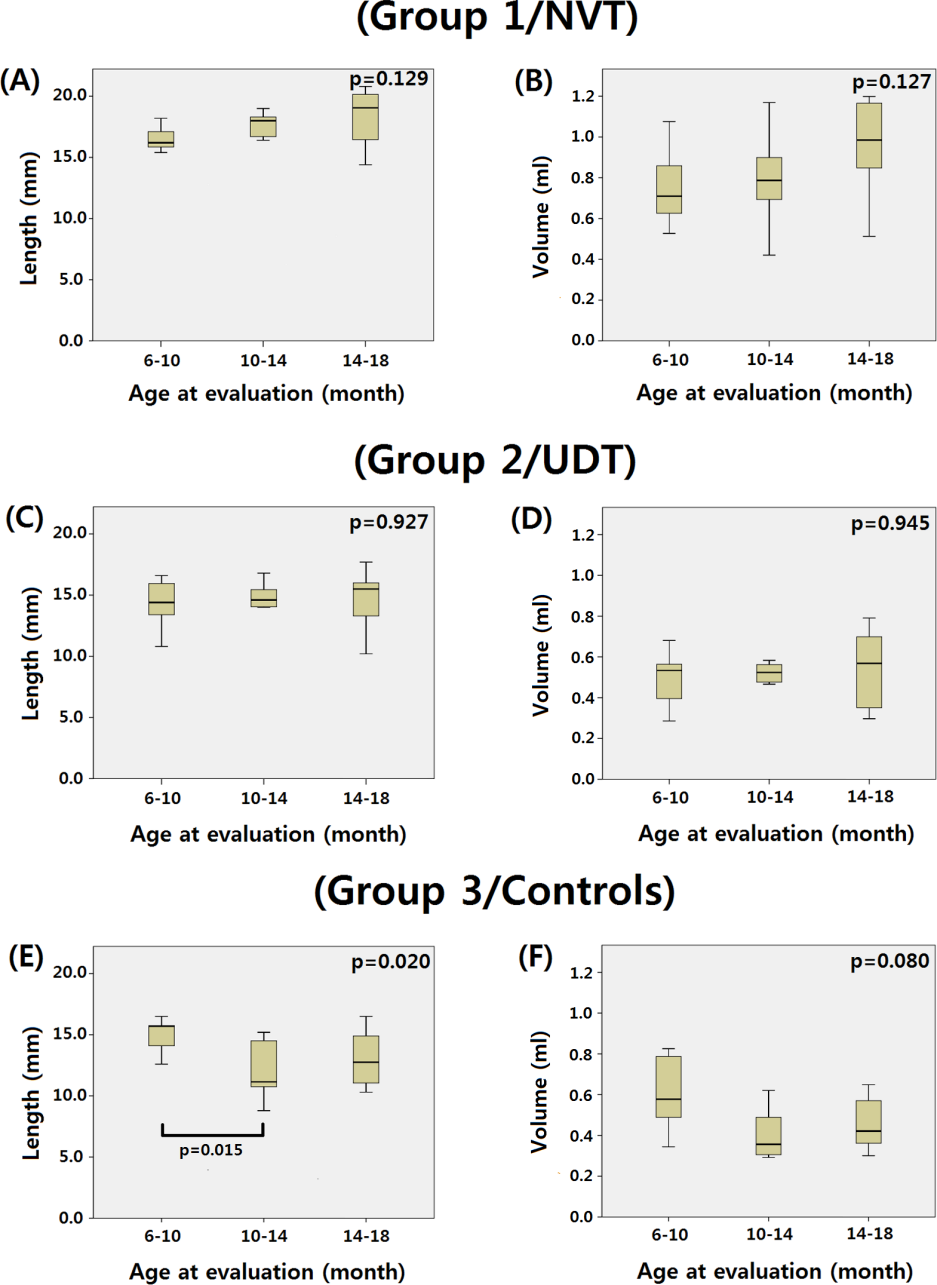
The variation of testicular length and volume during infancy by the effect of mini-puberty was absent in both NVTs and UDTs. In group 1 (NVT) (A-B) and group 2 (UDT) (C-D), there was no significant difference in testicular length or volume during any of the three periods assessed. In group 3 (controls), the length of the testes was significantly longer in infants at 6–10 months than at 10–14 months (E). The median testicular volume of the control group in infants at 6–10 months was larger than at 10–14 months, though without statistical significance (F).

We additionally analyzed whether changes in testicular size were observed in each group between 6 and 18 months. In group 1 (NVT) and group 2 (UDT), there was no significant difference in testicular length or volume during the three time periods (Fig 2A–2D). However, in controls (group 3), the length of the testes was significantly longer in infants at 6–10 months than at 10–14 months (p = 0.015; Fig 2E). The median testicular volume of the control group in infants at 6–10 months was larger than at 10–14 months, though without statistical significance (Fig 2F). After a sensitivity analysis of the mean testicular length and volume of each of the 12 controls, we found the same pattern as with the 24 control testes.

### Determination of the cut-off value for testicular length and volume predictive of a non-viable contralateral testis

**Fig 3:**
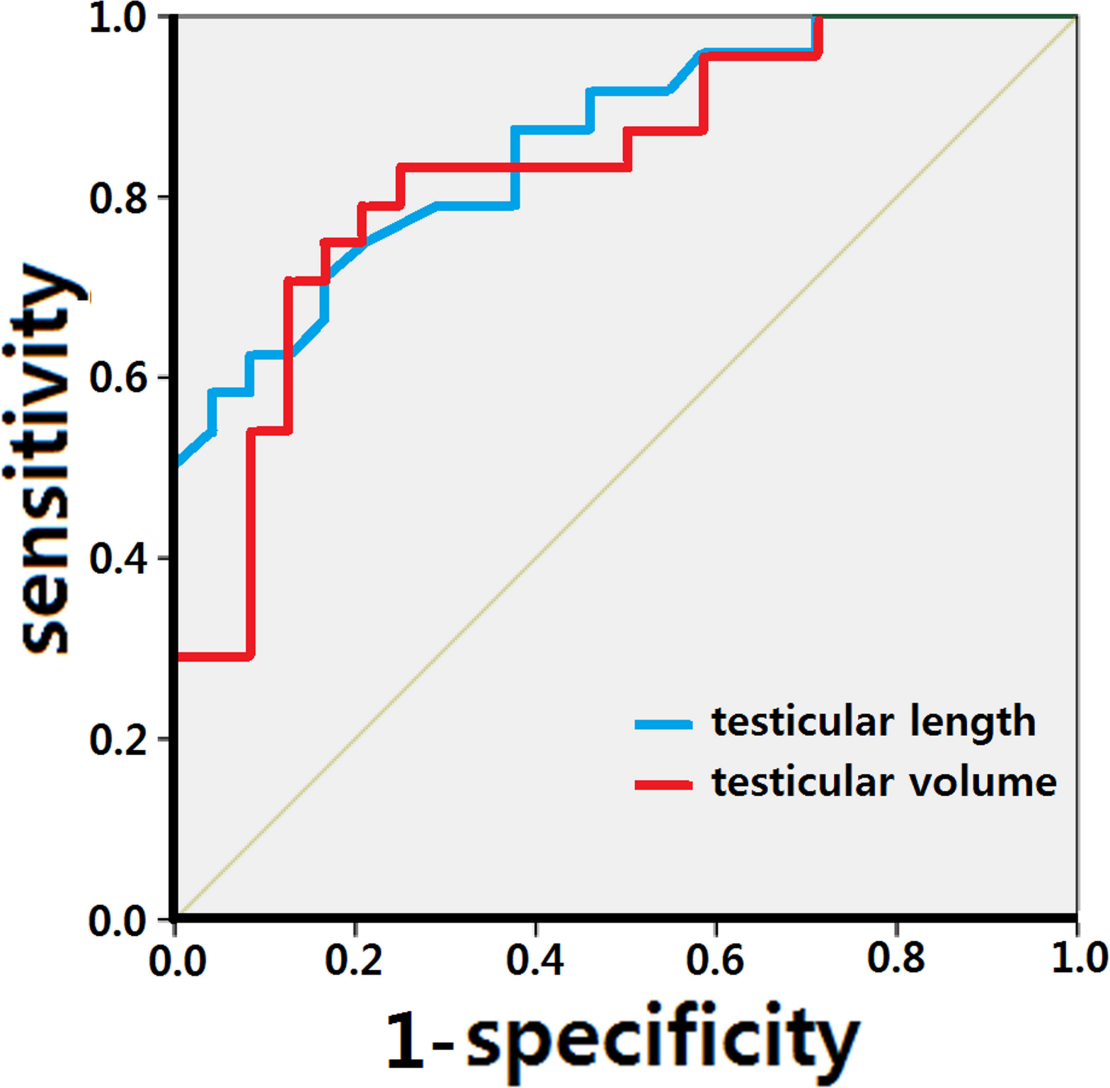
When testicular length was 16.1 mm (sensitivity 75.0%, specificity 79.2%) and volume was 0.59 ml (sensitivity 83.3%, specificity 75.0%), the combination of sensitivity and specificity was maximal. The area under the curve was calculated as 85.9% (95% confidence interval: 75.7%–96.2%) for testicular length and 83.3% (95% confidence interval: 71.8%–94.9%) for testicular volume.

To set the cut-off level for testicular length and volume predicting a NVT, the sensitivity and specificity for each length and volume was calculated among the 48 testes of group 1 (NVT) and group 2 (UDT). When the testicular length was 16.1 mm (sensitivity 75.0%, specificity 79.2%) or volume was 0.59 ml (sensitivity 83.3%, specificity 75.0%), the combination of sensitivity and specificity was maximal. We constructed a ROC curve based on these values (Fig 3). The area under the curve was calculated as 85.9% with testicular length and 83.3% with testicular volume.

## Discussion

Previously, the optimal cut-off value for prediction of a NVT was 20.0 mm in length and 2.0 ml in volume based on report by Koff et al. (Table 2).[3] Recently, Braga et al. proposed 19.0–20.0 mm in length.[6] Both the recommended testicular length and volume cut-offs in these studies were larger than the values in our study (16.1 mm for length and 0.59 ml for volume). There are a few differences between the previous reports and our study, including the method of measurement, age at evaluation, and patient grouping for calculating cut-off values. First, the discrepancy in volume was more prominent than that in length. Also, volume was not measured in three dimensions, or the information was not sufficient in previous reports. Koff measured the volume directly using an orchidometer, or indirectly calculated from the orchidometer by comparing the length of the long axis of the testis with the long axis of an orchidometer template of known testicular volume.[3] Shibata et al. used a caliper, but the method of volume measurement was not written in their report.[5] In contrast, the volume was measured in three dimensions in our study using ultrasonography. Kuijper et al. reported that the testes at early ages are not equivalent in shape; therefore, measurement in one plane may not represent the true volume.[9] Moreover, Sakamoto et al. demonstrated that a testicular ultrasound is more accurate than an orchidometer in assessing the testicular volume without overestimation.[13] Ultrasound can also exclude the inappropriate inclusion of the epididymis, scrotal skin, and adjacent soft tissue during the measurement.[14, 15] From this point of view, our study may achieve a greater precision than previous cut-off values of volume. A discrepancy in the age at evaluation could be a fundamental cause for differences between our findings and other published studies. Our study included the youngest group of patients among all the reports. Even a few months of age difference could result in a significant difference in testicular length and volume at this age. A proper cut-off level should be applied according to the patient age, especially in infancy. Nevertheless, the previous studies that calculated cut-off values had investigated heterogeneous patients with broad ranges of ages. This difference seems to be the reason why our cut-off values are different from theirs.

**Table 2:** Cut-off level for testicular length and volume reported previously.

	Included	Age at evaluation	Measurement methods		Cut-off value	
			Length	Volume	Length	Volume
Koff (1991)[3]	12 NVT	8 months-3years	Caliper or ruler	Takihara orchidometer or conversion of length into volume	20 mm	2.0 ml
	25 UDT					
Hurwitz and Kaptein (2001)[2]	40 NVT	7 month-11years	Conversion of volume to length and/or ruler	Takihara orchidometer	18 mm	N/A
	16 UDT					
Snodgrass et al. (2007)[11]	28 NVT	Median 23 (6–154) months	Ruler	N/A	18 mm	N/A
	12 UDT					
Shibata et al. (2010)[5]	33 NVT	Mean 19.4 (8–46) months	Caliper	N/A	22.4 mm	2.2 ml
	22 UDT					
Braga et al. (2014)[6]	35 NVT	Mean 28.0 ± 17.6 months	Caliper	Not measured	19–20 mm	N/A
	50 UDT					
Hodhod et al.(2015)[12]	46 NVT	Median 19.7 (8.4–109.4) months	N/A	Takihara orchidometer	N/A	2.0 ml
	26 UDT					
Current study	24 NVT	11.5 (IQR: 8.8–14.4) months	Ultrasonography	Ultrasonography	16.1 mm	0.59 ml
	24 UDT					

NVT: non-viable testis, UDT: viable undescended testis, N/A: not available, IQR: inter-quartile range

In this study, we calculated the cut-off level among 48 testes of non-palpable or palpable inguinal testes. In contrast, previous reports only included non-palpable testes in the analysis of cut-off values. In our study, 24 testes were proven to be NVTs among 31 preoperative non-palpable testes. We calculated the cut-off level among 48 testes, including 24 non-palpable and non-viable testes, 7 non-palpable and viable testes, and 17 palpable and viable testes. In contrast, Braga et al. included only preoperative non-palpable testes. However, among 85 non-palpable testes in their study, the proportion of NVTs was only 35. In the study by Koff et al., the authors similarly limited their inclusion criteria to only preoperative non-palpable testes, and only subsequently discovered 12 surgically proven NVTs. Therefore, a total of 120 NVTs and 113 UDTs were analyzed in the calculation of the recommended cut-off levels in the previous four reports. In conclusion, the composition of 24 NVTs and 24 UDTs in our study seemed similar to the previous reports, even though we included palpable testes.

Serum FSH, luteinizing hormone (LH), and testosterone levels begin to increase during the second week after birth, reaching a maximum level at approximately 4–10 weeks, and then decline by approximately 6 months of age to a low pre-pubertal level until the onset of puberty.[10, 16] This hormonal surge is the result of a transient spurt in the hypothalamic gonadotropin-releasing hormone pulse generator-pituitary gonadotropin-gonadal apparatus within a few minutes after birth, after being freed from the estrogenic inhibitory effect.[16] In this period, Sertoli cells are stimulated by FSH to produce a protein called Inhibin B.[17] In normal infants, the serum level of Inhibin B increases as a response to the incremental increase in serum FSH level and the resultant expansion in Sertoli cell numbers.[18, 19] Inhibin B controls the level of FSH secretion by a negative feedback mechanism. The serum level of Inhibin B remains elevated longer than the neonatal surge of FSH, LH, and testosterone, and is present at a detectable level until 15 months of age.[10] In early infancy, the testes are mainly composed of Sertoli cells, and the increase in testicular volume is mainly associated with an increase in seminiferous tubule length due to the proliferation of Sertoli cells stimulated by FSH.[20] Associated with the aforementioned fluctuation of hormonal levels or so-called ‘mini-puberty,’ testicular volume may also change. Kuijper et al. measured testicular volume with ultrasonography in normal children aged 0 to 6 years.[9] They revealed that testicular volume increases from birth to 5 months of age. Following this period, the volume declines and reaches a minimum level at approximately 9 months of age, with no further distinct size changes until 6 years of age. Our results in the control group are consistent with those of Kuijper et al.

Nevertheless, this effect of a ‘mini-puberty’ in our study was not observed in patients with a NVT or UDT. In the children with a NVT, even though the early activation of the hypothalamic-pituitary-gonadal hormone axis is intact, the decreased number of germ cells and Sertoli cells of the affected testes may induce a decreased total serum level of Inhibin B.[18] The relatively lower negative feedback may induce an unimpeded rise in the FSH level, which influences the growth of the seminiferous tubules in the contralateral testis, causing the resultant testicular enlargement. We observed a gradual increase in testicular size from the normal control group to the NVT group. This difference may have been due to the difference in the amount of testicular tissue affected. These observations corresponded to the findings reported by previous studies, demonstrating a correlation between the degree of the defect in the affected testis and the extent of CTH.[2–4]

The CTH related to the affected testicular volume could be also applied to the UDT group, along with another possible mechanism. Unlike with an NVT, a UDT could be related to a defect in the LH-Leydig cell axis. Several studies have proven that the levels of LH and testosterone between 1–4 months of age are significantly lower than normal in children with UDTs.[21–25] Previously, it was reported that the surge of plasma gonadotropins and testosterone in children with UDTs is lower than that of children with normal testes.[21, 26, 27] This phenomenon could be applied in the UDT group before 6 months of age. Although our results did not achieve statistical significance, the median value of testicular length and volume were smaller in group 2 (UDT) than in group 3 (control) at 6–10 months (Table 1). Following this period, a decreased number of Sertoli cells in the UDT group might have produced a smaller amount of negative feedback and caused the testes to be larger after 10 months of age.

One limitation of our study lies in the absence of corresponding data about plasma gonadotropins, Inhibin B, and testosterone to explain whether hormonal effects were actually present or not. Another limitation is an insufficient number of patients for the analysis of the testicular size variation according to the subdivided age groups, as our initial primary end-point was to compare testicular size among the three groups rather than to make subdivisions by age. In addition, to decrease the interobserver variability, measurements in this study were performed by single investigator. Further evaluation by multiple investigators is mandatory.

Nevertheless, we analyzed whether CTH is also effective during 6–18 months of age, the most important period in clinical practice according to recent guidelines. CTH was validated even in this period, but with different cut-off values. Moreover, a subgroup analysis revealed the absence of the effect of the so-called ‘mini-puberty’ on testicular volume in the NVT group for the first time, to our knowledge. Ultrasonographic measurement in our study could also be helpful for the correct measurement, as mentioned above. Further study with endocrinological evidence and serial follow-up evaluation on the testicular size could yield more information.

## Conclusions

The CTH in patients with an NVT or a UDT is also valid as a predictive tool for a non-viable testis even at 6–18 months after birth, as the effect of mini-puberty is absent in these patient groups. However, the cut-off values for testicular length and volume predicting the absence of viable testes are less than those of previous reports with older patients. Applying the proper cut-off level according to the age and measurement method is necessary, especially in infancy.

## Supporting Information

S1 File We attached our data sets. (XLSX)

S1 Table We performed a sensitivity analysis including a *post hoc* analysis with Bonferroni correction. After a sensitivity analysis of the mean testicular length and volume of each of the 12 controls, we got the same pattern as the results when using the 24 control testes.
(DOCX)

## References

[1] Laron Z, Dickerman Z, Ritterman I, Kaufman H. Follow-up of boys with unilateral compensatory testicular hypertrophy. Fertil Steril. 1980;33(3):297–301. Epub 1980/03/01. . 6102528 10.1016/s0015-0282(16)44598-x

[2] Hurwitz RS, Kaptein JS. How well does contralateral testis hypertrophy predict the absence of the nonpalpable testis? J Urol. 2001;165(2):588–92. Epub 2001/02/15. 10.1097/00005392-200102000-00077 . 11176443

[3] Koff SA. Does compensatory testicular enlargement predict monorchism? J Urol. 1991;146(2 (Pt 2)):632–3. Epub 1991/08/01. . 1677690 10.1016/s0022-5347(17)37877-1

[4] Huff DS, Snyder HM 3rd, Hadziselimovic F, Blyth B, Duckett JW. An absent testis is associated with contralateral testicular hypertrophy. J Urol. 1992;148(2 Pt 2):627–8. Epub 1992/08/01. . 1353543 10.1016/s0022-5347(17)36673-9

[5] Shibata Y, Kojima Y, Mizuno K, Nakane A, Kato T, Kamisawa H, et al Optimal cutoff value of contralateral testicular size for prediction of absent testis in Japanese boys with nonpalpable testis. Urology. 2010;76(1):78–81. Epub 2010/05/18. 10.1016/j.urology.2010.02.043 . 20472272

[6] Braga LH, Kim S, Farrokhyar F, Lorenzo AJ. Is there an optimal contralateral testicular cut-off size that predicts monorchism in boys with nonpalpable testicles? Journal of pediatric urology. 2014;10(4):693–8. Epub 2014/07/11. 10.1016/j.jpurol.2014.05.011 . 25008806

[7] Ritzen EM, Bergh A, Bjerknes R, Christiansen P, Cortes D, Haugen SE, et al Nordic consensus on treatment of undescended testes. Acta paediatrica (Oslo, Norway: 1992). 2007;96(5):638–43. Epub 2007/03/01. 10.1111/j.1651-2227.2006.00159.x . 17326760

[8] Kolon TF, Herndon CD, Baker LA, Baskin LS, Baxter CG, Cheng EY, et al Evaluation and treatment of cryptorchidism: AUA guideline. J Urol. 2014;192(2):337–45. Epub 2014/05/27. 10.1016/j.juro.2014.05.005 . 24857650

[9] Kuijper EA, van Kooten J, Verbeke JI, van Rooijen M, Lambalk CB. Ultrasonographically measured testicular volumes in 0- to 6-year-old boys. Hum Reprod. 2008;23(4):792–6. Epub 2008/02/19. 10.1093/humrep/den021 . 18281246

[10] Andersson AM, Toppari J, Haavisto AM, Petersen JH, Simell T, Simell O, et al Longitudinal reproductive hormone profiles in infants: peak of inhibin B levels in infant boys exceeds levels in adult men. The Journal of clinical endocrinology and metabolism. 1998;83(2):675–81. Epub 1998/02/19. 10.1210/jcem.83.2.4603 . 9467591

[11] Snodgrass WT, Yucel S, Ziada A. Scrotal exploration for unilateral nonpalpable testis. J Urol. 2007;178(4 Pt 2):1718–21. Epub 2007/08/21. 10.1016/j.juro.2007.05.089 . 17707015

[12] Hodhod A, Capolicchio JP, Jednak R, El-Sherbiny M. Testicular hypertrophy as a predictor for contralateral monorchism: Retrospective review of prospectively recorded data. Journal of pediatric urology. 2015 Epub 2015/08/19. 10.1016/j.jpurol.2015.06.010 . 26279100

[13] Sakamoto H, Saito K, Oohta M, Inoue K, Ogawa Y, Yoshida H. Testicular volume measurement: comparison of ultrasonography, orchidometry, and water displacement. Urology. 2007;69(1):152–7. Epub 2007/02/03. 10.1016/j.urology.2006.09.012 . 17270639

[14] Lin CC, Huang WJ, Chen KK. Measurement of testicular volume in smaller testes: how accurate is the conventional orchidometer? Journal of andrology. 2009;30(6):685–9. Epub 2009/07/07. 10.2164/jandrol.108.006460 . 19578133

[15] Sakamoto H, Saito K, Ogawa Y, Yoshida H. Testicular volume measurements using Prader orchidometer versus ultrasonography in patients with infertility. Urology. 2007;69(1):158–62. Epub 2007/02/03. 10.1016/j.urology.2006.09.013 . 17270640

[16] Grumbach MM. A window of opportunity: the diagnosis of gonadotropin deficiency in the male infant. The Journal of clinical endocrinology and metabolism. 2005;90(5):3122–7. Epub 2005/02/25. 10.1210/jc.2004-2465 . 15728198

[17] Crofton PM, Evans AE, Groome NP, Taylor MR, Holland CV, Kelnar CJ. Inhibin B in boys from birth to adulthood: relationship with age, pubertal stage, FSH and testosterone. Clinical endocrinology. 2002;56(2):215–21. Epub 2002/03/05. . 11874413 10.1046/j.0300-0664.2001.01448.x

[18] Thorup J, Kvist K, Clasen-Linde E, Hutson JM, Cortes D. Serum Inhibin B Values in Boys with Unilateral Vanished Testis or Unilateral Cryptorchidism. J Urol. 2014 Epub 2014/12/03. 10.1016/j.juro.2014.10.110 . 25451827

[19] Main KM, Toppari J, Suomi AM, Kaleva M, Chellakooty M, Schmidt IM, et al Larger testes and higher inhibin B levels in Finnish than in Danish newborn boys. The Journal of clinical endocrinology and metabolism. 2006;91(7):2732–7. Epub 2006/04/06. 10.1210/jc.2005-2443 . 16595596

[20] Muller J, Skakkebaek NE. Quantification of germ cells and seminiferous tubules by stereological examination of testicles from 50 boys who suffered from sudden death. International journal of andrology. 1983;6(2):143–56. Epub 1983/04/01. . 6862671 10.1111/j.1365-2605.1983.tb00333.x

[21] Hamza AF, Elrahim M, Elnagar, Maaty SA, Bassiouny E, Jehannin B. Testicular descent: when to interfere? Eur J Pediatr Surg. 2001;11(3):173–6. Epub 2001/07/28. 10.1055/s-2001-15484 . 11475113

[22] Job JC, Toublanc JE, Chaussain JL, Gendrel D, Garnier P, Roger M. Endocrine and immunological findings in cryptorchid infants. Horm Res. 1988;30(4–5):167–72. Epub 1988/01/01. . 2907894 10.1159/000181055

[23] Gendrel D, Roger M, Job JC. Plasma gonadotropin and testosterone values in infants with cryptorchidism. J Pediatr. 1980;97(2):217–20. Epub 1980/08/01. . 6105174 10.1016/s0022-3476(80)80477-x

[24] De Muinck Keizer-Schrama SM, Hazebroek FW, Drop SL, Degenhart HJ, Molenaar JC, Visser HK. Hormonal evaluation of boys born with undescended testes during their first year of life. The Journal of clinical endocrinology and metabolism. 1988;66(1):159–64. Epub 1988/01/01. 10.1210/jcem-66-1-159 . 2891719

[25] Suomi AM, Main KM, Kaleva M, Schmidt IM, Chellakooty M, Virtanen HE, et al Hormonal changes in 3-month-old cryptorchid boys. The Journal of clinical endocrinology and metabolism. 2006;91(3):953–8. Epub 2006/01/06. 10.1210/jc.2004-2318 . 16394094

[26] Raivio T, Toppari J, Kaleva M, Virtanen H, Haavisto AM, Dunkel L, et al Serum androgen bioactivity in cryptorchid and noncryptorchid boys during the postnatal reproductive hormone surge. The Journal of clinical endocrinology and metabolism. 2003;88(6):2597–9. Epub 2003/06/06. 10.1210/jc.2002-021676 . 12788861

[27] Bouvattier C, Carel JC, Lecointre C, David A, Sultan C, Bertrand AM, et al Postnatal changes of T, LH, and FSH in 46,XY infants with mutations in the AR gene. The Journal of clinical endocrinology and metabolism. 2002;87(1):29–32. Epub 2002/01/15. 10.1210/jcem.87.1.7923 . 11788616

