# Supplementary material for: Can Hypertrophy of the Contralateral Testis Predict the Absence of a Viable Testis in Infancy with Cryptorchidism: A Prospective Analysis
Source: PLoS One. 2016 Mar 18;11(3):e0151528. doi: 10.1371/journal.pone.0151528 (PMC4798213; doi:10.1371/journal.pone.0151528)
Supplement: S1 Table — After a sensitivity analysis of the mean testicular length and volume of each of the 12 controls, we got the same pattern as the results when using the 24 control testes. (DOCX) [file pone.0151528.s002.docx]

We performed a sensitivity analysis along with a *post hoc* analysis with Bonferroni correction. After a sensitivity analysis of the mean testicular length and volume of each of the 12 controls, we got the same pattern as our previous results when using the 24 control testes.

|  |  |  | | Post-hoc with “Bonferroni correction” (p-value) | | |
| --- | --- | --- | --- | --- | --- | --- |
|  |  | Pattern | p-value | 1 vs 2 | 2 vs 3 | 3 vs 1 |
| 24 control testes | Figure 1(A) | 1>2>3 | <0.001 | <0.001 | 0.219 | <0.001 |
|  | Figure 1(B) | 1>2>3 | <0.001 | <0.001 | >0.999 | <0.001 |
|  | Figure 2(E) | 6-10>14-18>10-14 | 0.020 | 0.015 | >0.999 | 0.150 |
|  | Figure 2(F) | 6-10>14-18>10-14 | 0.080 | N/A | N/A | N/A |
| 12 control testes | Figure 1(A) | 1>2>3 | <0.001 | <0.001 | 0.336 | <0.001 |
|  | Figure 1(B) | 1>2>3 | <0.001 | <0.001 | >0.999 | <0.001 |
|  | Figure 2(E) | 6-10>14-18>10-14 | 0.092 | N/A | N/A | N/A |
|  | Figure 2(F) | 6-10>14-18>10-14 | 0.167 | N/A | N/A | N/A |
